# Prognostic value of easy Albumin-Bilirubin score in liver cirrhosis: a comparison with established scoring systems

**DOI:** 10.1097/MEG.0000000000003118

**Published:** 2025-12-09

**Authors:** Jing Liu, Yi-sheng Wei

**Affiliations:** 1 Department of Gastroenterology, The First People’s Hospital of Xianyang, Xianyang, China

**Keywords:** easy-Albumin-Bilirubin score, liver cirrhosis, prognosis, scoring systems, survival prediction

## Abstract

**Background and aims:**

Accurate prognostication critical for managing liver cirrhosis. Existing tools [Child-Pugh, model for end-stage liver disease (MELD), MELD-sodium (MELD-Na), Albumin-Bilirubin (ALBI), and platelet ALBI (PALBI)] have limitations, including subjectivity, complexity, and reliance on logarithmic transformations. Simplified easy-ALBI (EZ-ALBI), a prognostic factor in hepatocellular carcinoma, is understudied in cirrhosis. The present study aimed to evaluate its prognostic value in cirrhosis, compare it with established scores, assess its consistent long-term performance, and define its clinical utility for risk stratification.

**Methods:**

This retrospective study enrolled 501 cirrhotic patients (June 2018–June 2020), with a median follow-up of 42.3 months (interquartile range: 28.6–56.8 months); follow-up was terminated on 30 June 2025. EZ-ALBI was compared with Child-Pugh, MELD-Na, ALBI, and PALBI using correlation, survival (Kaplan–Meier), Cox regression, and receiver operating characteristic analyses.

**Results:**

EZ-ALBI strongly correlated with ALBI (*r* = 0.9460, *P* < 0.001). EZ-ALBI grade 3 was associated with shorter survival (29.9 vs. 65.5 months, *P* < 0.001) and served as an independent prognostic factor (hazard ratio = 3.944, 95% confidence interval: 1.772–8.777, *P* < 0.05). Its prognostic accuracy was consistent across 6–60 months (areas under the curves: 0.738–0.832), long-term performance (36–60 months) was comparable to MELD, MELD-Na, and ALBI, and outperformed Child-Pugh and PALBI in specific periods.

**Conclusion:**

The EZ-ALBI score is a simple, objective, and reliable prognostic tool for patients with liver cirrhosis, with consistent predictive value across follow-up periods, supporting its clinical utility for risk stratification. Notably, EZ-ALBI’s simplicity (no logarithmic transformations) significantly enhances its practicality for bedside risk stratification, a key advantage in clinical practice.

## Introduction

Liver cirrhosis, a major global health burden, is characterized by progressive hepatic fibrosis, impaired synthetic function, and portal hypertension, and frequently progresses to decompensation (e.g. ascites, variceal bleeding, and encephalopathy) [1,2]. With rising global incidence and high mortality, particularly in decompensated stages (median survival: 2 years; 5-year survival: approximately 45%) [3,4], accurate prognostic stratification is pivotal for guiding clinical decisions and improving outcomes [2,5].

Over the past decades, multiple scoring systems have been developed for assessing disease severity and predicting survival in patients with cirrhosis. Among the most widely used for cirrhosis prognosis, these systems fall into three categories: subjective indices (e.g. Child-Pugh), those incorporating renal function [e.g. model for end-stage liver disease (MELD), MELD-sodium (MELD-Na)], and those focusing on liver synthetic function [e.g. Albumin-Bilirubin (ALBI), platelet ALBI (PALBI)]. The Child-Pugh score integrates clinical parameters (ascites, encephalopathy) and biochemical parameters, but is limited by subjective assessments and coarse grading [6–8]. MELD and MELD-Na, as objective alternatives that use bilirubin, creatinine, international normalized ratio (INR), and sodium, excel in short-term transplant prognostication, but the complexity of MELD-Na limits its long-term utility in resource-limited settings [9–13].

More recently, the ALBI score has attracted attention due to its simplicity and objectivity, as it uses only serum albumin and bilirubin to assess hepatic reserve [14]. Its derived indices, such as the PALBI score (which incorporates platelet count to reflect portal hypertension) and the easy ALBI (EZ-ALBI) score (which eliminates logarithmic transformations for bedside use), have further expanded the range of prognostic tools for liver disease [15–17]. Unlike ALBI, the EZ-ALBI score [calculated as total bilirubin (mg/dl) min 9 × albumin (g/dl)] eliminates ALBI’s logarithmic transformations of ALBI, thereby enhancing bedside utility. Initially validated for hepatocellular carcinoma (HCC) [16–18], it may be extendable to cirrhosis owing to shared core features of hepatic dysfunction, serving as a candidate prognostic tool for both conditions. However, its prognostic value in cirrhosis, particularly in non-HCC populations and across long-term follow-up, remains understudied, and no systematic comparisons to established scoring systems have been made; this critical gap hindering its clinical translation.

In this retrospective study, we aimed to systematically evaluate the utility of the EZ-ALBI as a prognostic marker in cirrhotic patients. Specifically, we aimed to: (1) assess the correlation between EZ-ALBI and established scores (Child-Pugh, MELD, MELD-Na, ALBI, and PALBI); (2) determine whether EZ-ALBI grade independently predicts survival outcomes; and (3) compare the prognostic accuracy of EZ-ALBI with other models for short-term (6, 12 months) and long-term (36, 60 months) survival, given differing short- and long-term prognostic needs in clinical practice. We hypothesized that the EZ-ALBI score, due to its simplicity and reliance on key synthetic liver function markers, would serve as a robust and clinically feasible tool for risk stratification in cirrhosis.

## Patients and methods

### Patients and research methods

#### Study population

Patients initially diagnosed with liver cirrhosis admitted to the Department of Gastroenterology, the First People’s Hospital of Xianyang, from June 2018 to June 2020 were retrospectively enrolled. Clinical data of eligible patients were collected from the hospital’s electronic medical records.

#### Data collection

Collected data included:

1. Baseline characteristics: age, gender, and etiology of cirrhosis.
2. Laboratory parameters: serum albumin, total bilirubin, alanine aminotransferase (ALT), aspartate aminotransferase (AST), platelet count, neutrophil count, serum sodium (Na), and INR. Prognostic scores (Child-Pugh, MELD, MELD-Na, ALBI, PALBI, and EZ-ALBI) were calculated via established formulas and grading criteria [6,7,9,12,16,17]. Specifically, the EZ-ALBI score was calculated as: total bilirubin (mg/dl) − 9×albumin (g/dl), with grading: 1: ≤−34.4; 2: −34.4 to −22.2; 3: ≥−22.2 [16,17].
3. Imaging findings: radiological and endoscopic assessments.
4. Clinical course: complications, therapeutic interventions, and follow-up outcomes.

#### Follow-up

Patients’ survival status was monitored via electronic medical record reviews and regular telephone follow-ups conducted by trained gastroenterologists, starting 1 month postenrollment, then every 3–6 months. Patients were defined as lost to follow-up if they were unreachable for >6 consecutive months despite ≥3 attempts. The median follow-up duration was 42.3 months [interquartile ranges (IQR): 28.6–56.8 months], with detailed records:

1. Survival status: complications (e.g. ascites, bleeding) and treatment adjustments.
2. Survival time: from initial cirrhosis diagnosis to death or follow-up censoring (30 June 2025).
3. Causes of death: HCC, liver failure, gastrointestinal bleeding, severe infection, and hepatorenal syndrome.
4. Loss to follow-up: dates and potential reasons for lost patients.

#### Inclusion and exclusion criteria

##### Inclusion criteria

Patients with liver cirrhosis who met the diagnostic criteria specified in the Chinese Guidelines for the Management of Liver Cirrhosis (2019 Edition) [19].

##### Exclusion criteria

1. Age <18 or >80 years.
2. Severe primary cardiopulmonary or renal diseases [e.g. heart failure, respiratory failure, chronic kidney disease (stage ≥3)].
3. Concomitant malignant tumors other than primary liver cancer during the study.
4. Incomplete clinical data or loss to follow-up.

#### Study cohort

**Fig. 1.**
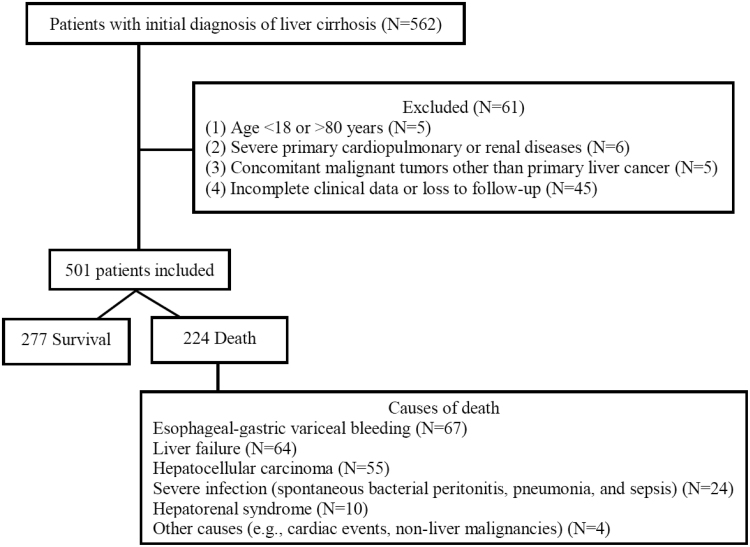
Flowchart of 501 cirrhosis patient’s enrollment (June 2018–June 2020) and outcomes (follow-up ended 30 June 2025).

Initial screening identified newly diagnosed cirrhotic patients from June 2018 to June 2020. After applying exclusion criteria (incomplete data, loss to follow-up, or age outside the specified range), 501 patients were included, exceeding the required sample size of 450. By follow-up end, 277 patients were alive, and 224 had died (enrollment flow is detailed in Fig. 1; exclusions were primarily due to incomplete data and loss to follow-up).

### Statistical analysis

The sample size was determined based on a previous pilot study showing a 2-year mortality rate of ~30% in cirrhotic patients, with a power of 90% and α = 0.05, at least 450 cases were required. Analysis was conducted using IBM SPSS version 28.0. Normality of continuous variables was assessed using the Shapiro–Wilk test combined with Q–Q plots. Nonnormally distributed continuous variables were presented as medians (IQR) and analyzed via the Mann–Whitney *U* test. Categorical variables are reported as frequencies (%) and compared using the *χ*² test or Fisher’s exact test. Kaplan–Meier survival curves and log-rank tests were utilized to compare survival rates between groups. The Youden index determined optimal cutoffs for prognostic factors, which were analyzed via Cox regression. Predictive accuracy was assessed using receiver operating characteristic (ROC) curves, and DeLong’s test was used to compare the areas under the ROC curves (AUROCs). A two-tailed *P* < 0.05 was considered statistically significant.

### Ethical statement

The Ethics Committee of the First People’s Hospital of Xianyang approved this retrospective study (approval number XYSDYRMYY-KYLC-010416001) and waived informed consent due to patient data anonymization.

## Results

### Correlation analysis

**Fig. 2.**
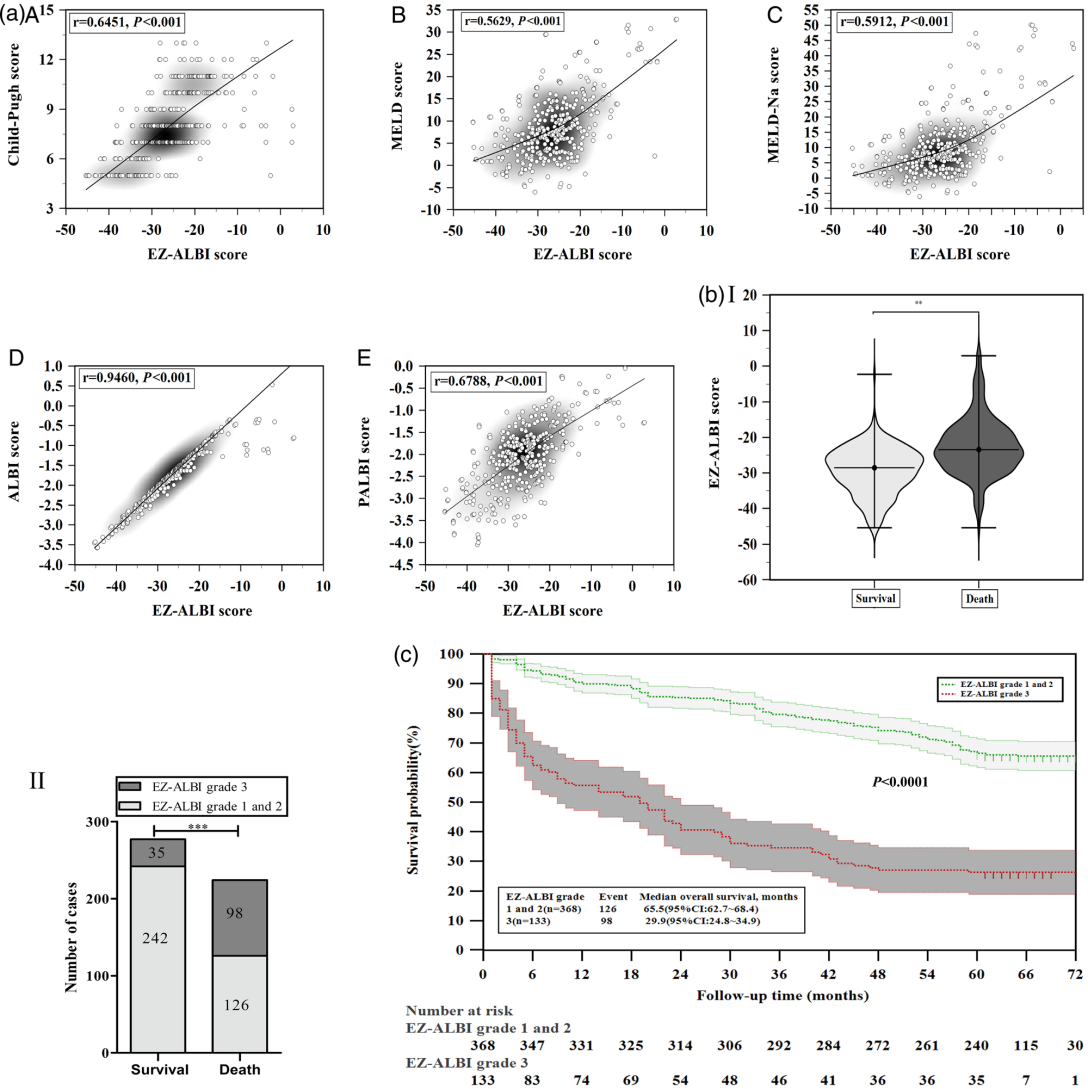
Correlation and prognostic value of easy Albumin-Bilirubin (EZ-ALBI) score in liver cirrhosis. (a) A–E: Correlations between EZ-ALBI and other scores (all *P* < 0.001). (b) EZ-ALBI scores (I) and grades (II) in survival vs. death groups. (c) Kaplan–Meier curves by EZ-ALBI grades (grade 3 vs. 1–2, *P* < 0.001).

As shown in Fig. 2a, the EZ-ALBI score showed significant positive correlations with the Child-Pugh (*r = *0.6451, *P < *0.001), MELD (*r = *0.5629, *P < *0.001), and MELD-Na (*r* = 0.5912, *P* < 0.001). Notably, it showed an extremely strong correlation with the ALBI (*r = *0.9460, *P* < 0.001) and a relatively strong correlation with the PALBI (*r* = 0.6788, *P* < 0.001).

### Patient characteristics

Significant differences in key indicators were observed, with the death group showing higher total bilirubin (36.3 vs. 18.6 μmol/l, *Z* = 8.002, *P* < 0.0001), lower albumin (30.3 vs. 33.6 g/l, *Z* = 8.445, *P* < 0.0001), and poorer EZ-ALBI scores (−23.4 vs. −28.6, *Z* = 11.277, *P* < 0.0001) (Table 1, Fig. 2b I). The death group exhibited a higher proportion of EZ-ALBI grade 3 (43.7 vs. 12.6%, *χ*² = 61.366, *P* < 0.0001; Fig. 2b II). Notably, among the 224 patients in the death group, specific causes of death were as follows: esophageal-gastric variceal bleeding (67 cases, 29.9%), liver failure (64 cases, 28.6%), HCC (55 cases, 24.6%), severe infection (including SBP, pneumonia, and sepsis; 24 cases, 10.7%), hepatorenal syndrome (10 cases, 4.5%), and other causes (e.g. cardiac events, nonliver malignancies; four cases, 1.8%) (Fig. 1).

**Table 1.** Characteristics of patients with liver cirrhosis

Variable	Survival group (*n* = 277)	Death group (*n* = 224)	*χ*²/*Z*	*P* value	Overall (*n* = 501)
Sex (female/male), *n* (%)	136/141 (49.1/50.9)	106/118 (47.3/52.7)	*χ*² = 0.156	0.6927	242/259 (48.3/51.7)
Age (years)	57 (49–66)	65 (54–73)	*Z* = 5.655	<0.0001	60 (51–69)
Etiology (virus/others), *n* (%)	224/53 (80.9/19.1)	190/34 (84.8/15.2)	*χ*² = 1.347	0.2457	414/87 (82.6/17.4)
Platelets (10^9^/l)	64 (42–115)	57 (39–64)	*Z* = 1.838	0.0661	60 (40–96)
Total bilirubin (μmol/l)	18.6 (15.1–35.9)	36.3 (22.5–66.1)	*Z* = 8.002	<0.0001	27.1 (17–48.1)
Albumin (g/l)	33.6 (29.6–38.6)	30.3 (25.2–33.5)	*Z* = 8.445	<0.0001	31.6 (27.6–36.1)
Prothrombin time (s)	14 (12.7–15.7)	16.4 (14.5–20)	*Z* = 10.191	<0.0001	15 (13.3–17.4)
Cholinesterase (IU/l)	3516 (2743–4984)	2257 (1766–2906)	*Z* = 15.935	<0.0001	2912 (2136–3943)
Creatinine (μmol/l)	61 (50–76)	73.3 (55.3–101.1)	*Z* = 5.169	<0.0001	66 (51–83.1)
INR	1.19 (1.08–1.38)	1.39 (1.24–1.6)	*Z* = 9.882	<0.0001	1.27 (1.13–1.49)
Na (mmol/l)	141 (138.9–142.3)	138 (132.3–141)	*Z* = 7.930	<0.0001	140 (136–142)
Neutrophil count (10⁹/l)	2.12 (1.45–3.45)	2.85 (1.82–5.13)	*Z* = 4.334	<0.0001	2.38 (1.57–3.87)
AST (U/l)	33 (23–53)	37 (24–73)	*Z* = 2.891	0.0038	34 (24–60)
ALT (U/l)	24 (15–41)	26 (16–54)	*Z* = 1.761	0.0782	25 (16–44)
Total bile acids (μmol/l)	18.6 (9.6–34.1)	24.5 (11.3–57.3)	*Z* = 3.293	0.0010	21.4 (10.3–43.3)
Child-Pugh grade (A and B/C), *n* (%)	237/40 (85.6/14.4)	136/88 (60.7/39.3)	*χ*² = 40.111	<0.0001	373/128 (74.5/25.5)
MELD score	5.9 (2.2–9.5)	11.4 (6.4–15.9)	*Z* = 10.078	<0.0001	7.6 (3.7–13)
MELD-Na score	6.2 (2.2–9.6)	12.3 (6.8–22.6)	*Z* = 10.976	<0.0001	8 (3.8–14.3)
ALBI grade (1 and 2/3), *n* (%)	237/40 (85.6/14.4)	123/101 (54.9/45.1)	*χ²* = 57.412	<0.0001	360/141 (71.9/28.1)
PALBI grade (1 and 2/3), *n* (%)	158/119 (57/43)	67/157 (29.9/70.1)	*χ*² = 36.768	<0.0001	225/276 (44.9/55.1)
EZ-ALBI score	−28.6 (−33.6 to −24.7)	−23.4 (−28.4–18.4)	*Z* = 11.277	<0.0001	−26.1 (−30.8 to −21.9)
EZ-ALBI grade (1 and 2/3), *n* (%)	242/35 (87.4/12.6)	126/98 (56.2/43.7)	*χ*² = 61.366	<0.0001	368/133 (73.5/26.5)

Statistic (*χ²* for categorical variables/*Z* for continuous variables).

ALBI, Albumin-Bilirubin; ALT, alanine aminotransferase; AST, aspartate aminotransferase; EZ-ALBI, easy ALBI; INR, international normalized ratio; MELD, model for end-stage liver disease; MELD-Na, MELD-sodium; Na, serum sodium; PALBI, platelet ALBI.

### Survival curves of patients with liver cirrhosis stratified by easy Albumin-Bilirubin grade 3 vs. grades 1 and 2

Figure 2c displayed that the patients with EZ-ALBI grades 1 and 2 (*n* = 368) had a significantly longer median overall survival time [65.5 months, 95% confidence interval (CI): 62.7–68.4 months] compared with those with EZ-ALBI grade 3 (*n* = 133; median overall survival: 29.9 months, 95% CI: 24.8–34.9 months). The log-rank test showed a statistically significant difference in survival between the two groups (*P* < 0.001).

### Prognostic factors for liver cirrhosis: univariate and multivariate analyses

**Fig. 3.**
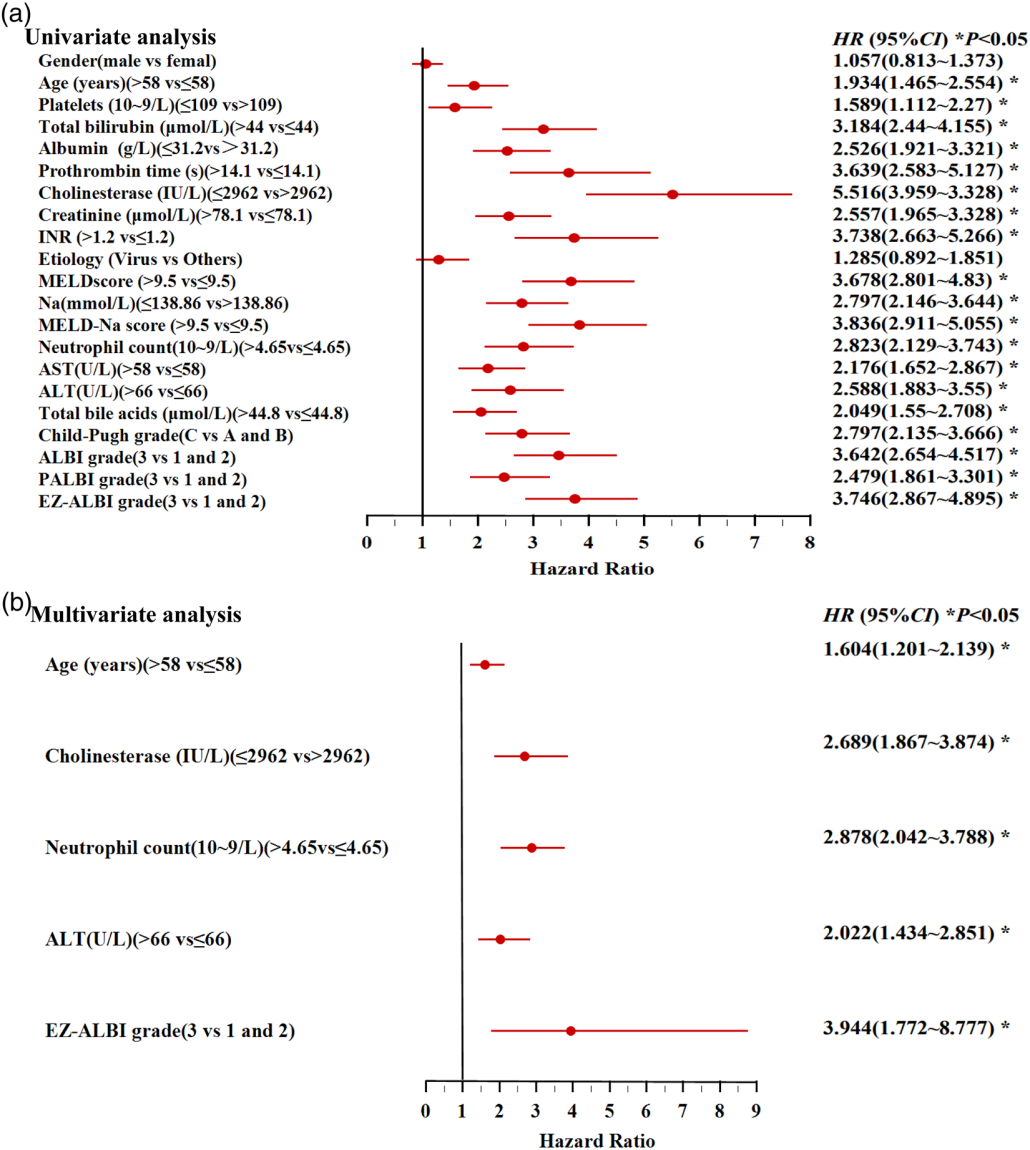
Prognostic factors for liver cirrhosis: (a) univariate and (b) multivariate analyses.

In the univariate analysis (Fig. 3a), multiple clinical factors were associated with survival, including sex, age, platelet count, total bilirubin, albumin, prothrombin time, cholinesterase, creatinine, INR, etiology, MELD, serum sodium (Na), MELD-Na, neutrophil count, AST, ALT, total bile acids, Child-Pugh, ALBI, PALBI, and EZ-ALBI. Among these factors, EZ-ALBI grade 3 (vs. grades 1 and 2) had a significant hazard ratio (HR = 3.746, 95% CI: 2.867–4.895, *P* < 0.05).

In the multivariate analysis (Fig. 3b), independent prognostic factors were:

1. Age (>58 vs. ≤58 years: HR = 1.604, 95% CI: 1.201–2.139, *P* < 0.05).
2. Cholinesterase (≤2962 vs. >2962 IU/l: HR = 2.689, 95% CI: 1.867–3.874, *P* < 0.05).
3. Neutrophil count (>4.65 vs. ≤4.65 × 10⁹/l: HR = 2.878, 95% CI: 2.042–3.788, *P* < 0.05).
4. ALT (>66 vs. ≤66 U/l: HR = 2.022, 95% CI: 1.434–2.851, *P* < 0.05).
5. EZ-ALBI grade 3 (vs. 1 and 2: HR = 3.944, 95% CI: 1.772–8.777, *P* < 0.05).

### Prognostic performance of various models for survival at 6, 12, 36, and 60 months

**Fig. 4.**
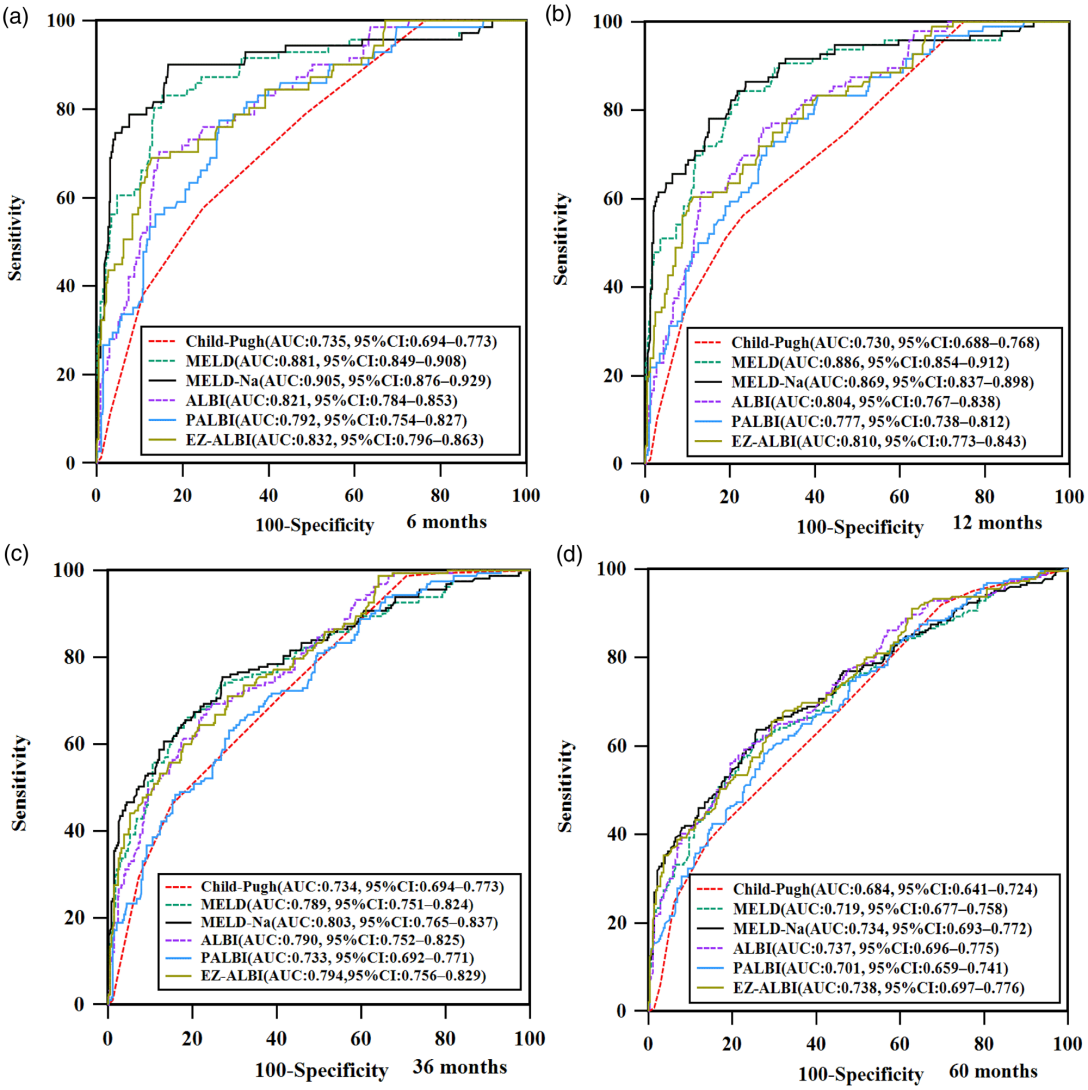
ROC curves comparing prognostic accuracy of EZ-ALBI vs. established systems for 6-, 12-, 36-, and 60-month survival in cirrhosis (a–d). AUC with 95% CI is shown for Child-Pugh, MELD, MELD-Na, ALBI, PALBI, and EZ-ALBI. ALBI, Albumin-Bilirubin; CI, confidence interval; EZ-ALBI, easy Albumin-Bilirubin; EZ-ALBI, easy-Albumin-Bilirubin; MELD, model for end-stage liver disease; MELD-Na, model for end-stage liver disease-sodium; PALBI, platelet Albumin-Bilirubin; ROC, receiver operating characteristic.

As illustrated in Fig. 4 (ROC curves) and Table 2 (AUROC comparisons), EZ-ALBI showed consistent prognostic accuracy across 6–60 months [areas under the curves (AUC): 0.738–0.832]. Notable differences from other models are as follows:

**Table 2.** AUC and 95% confidence intervals of different prognostic models at multiple time-points and their *P*-values compared with the easy-Albumin-Bilirubin model

Prognostic models	6 m AUC (95% CI)	*P* 6 m	12 m AUC (95% CI)	*P* 12 m	36 m AUC (95% CI)	*P* 36 m	60 m AUC (95% CI)	*P* 60 m
EZ-ALBI	0.832 (0.796–0.863)		0.810 (0.773–0.843)		0.794 (0.756–0.829)		0.738 (0.697–0.776)	
Child-Pugh	0.735 (0.694–0.773)	**0.0011**	0.730 (0.688–0.768)	**0.0011**	0.734 (0.694–0.773)	**0.0025**	0.684 (0.641–0.724)	**0.0046**
MELD	0.881 (0.849–0.908)	0.1385	0.886 (0.854–0.912)	**0.0353**	0.789 (0.751–0.824)	0.8526	0.719 (0.677–0.758)	0.4448
MELD-Na	0.905 (0.876–0.929)	**0.0231**	0.869 (0.837–0.898)	**0.0056**	0.803 (0.765–0.837)	0.7224	0.734 (0.693–0.772)	0.8735
ALBI	0.821 (0.784–0.853)	0.0713	0.804 (0.767–0.838)	0.2571	0.790 (0.752–0.825)	0.3009	0.737 (0.696–0.775)	0.7962
PALBI	0.792 (0.754–0.827)	0.0593	0.777 (0.738–0.812)	0.0948	0.733 (0.692–0.771)	**0.0025**	0.701 (0.659–0.741)	0.0832

ALBI, Albumin-Bilirubin; EZ-ALBI, easy-Albumin-Bilirubin; MELD, model for end-stage liver disease; MELD-Na, model for end-stage liver disease-sodium; PALBI, platelet Albumin-Bilirubin.

*P* < 0.05 vs. the EZ-ALBI score (boldfaced).

At 6 months, MELD-Na (AUC = 0.905, *P* = 0.0231) outperformed EZ-ALBI (AUC = 0.832), while Child-Pugh (AUC = 0.735, *P* = 0.0011) underperformed.

At 12 months, MELD (AUC = 0.886, *P* = 0.0353) and MELD-Na (AUC = 0.869, *P* = 0.0056) outperformed EZ-ALBI (AUC = 0.810), while Child-Pugh (AUC = 0.730, *P* = 0.0011) underperformed.

At 36 months, EZ-ALBI (AUC = 0.794) outperformed Child-Pugh (AUC = 0.734, *P* = 0.0025) and PALBI (AUC = 0.733, *P* = 0.0025).

Finally, at 60 months, EZ-ALBI (AUC = 0.738) outperformed Child-Pugh (AUC = 0.684, *P* = 0.0046).

## Discussion

Accurate prognostic assessment is critical for optimizing clinical management of cirrhotic patients. Liver cirrhosis remains a global health challenge with high morbidity and mortality, particularly in decompensated stages, which complications drive poor outcomes [1,2,20]. Although established scoring systems (e.g. Child-Pugh, MELD, ALBI) have aided in risk stratification, they are limited by subjectivity (Child-Pugh), computational complexity (MELD-Na), or variable performance across clinical scenarios (PALBI). This study systematically evaluated the prognostic utility of the EZ-ALBI score in cirrhotic patients by comparing it to established models (Child-Pugh, MELD, MELD-Na, ALBI, PALBI). Given these limitations of established models, our findings indicate that EZ-ALBI is a robust and practically valuable tool for risk stratification with unique advantages in simplicity and accuracy.

EZ-ALBI correlated positively with Child-Pugh (*r* = 0.6451), MELD (*r* = 0.5629), and MELD-Na (*r* = 0.5912), with the strongest correlation to ALBI (*r* = 0.9460, all *P* < 0.001). It retains ALBI’s ability to assess liver synthetic function (via albumin and bilirubin) but eliminates logarithms, thus facilitating bedside use [18,21]. This relevance arises from its alignment with cirrhosis pathophysiology, albumin reflects hepatic synthetic capacity, and bilirubin mirrors excretory function [2]. Notably, ALBI’s clinical relevance extends beyond survival prediction; a recent study showed that high ALBI scores at 12 weeks after sustained virological response (SVR) independently predict esophageal varices aggravation or de novo formation in cirrhotic patients [22], highlighting its utility in assessing portal hypertension-related complications. A systematic review and meta-analysis confirmed that ALBI grade is a robust prognostic indicator for liver disease (including postresection HCC) [23], supporting the clinical value of ALBI-derived indices like EZ-ALBI. A moderate correlation with PALBI (*r* = 0.6788, *P* < 0.001) reflects indirect capture of portal hypertension via bilirubin/albumin, consistent with the pathophysiological cascade where impaired liver synthetic function exacerbates portal hypertension, which in turn disrupts bilirubin clearance and albumin production. This aligns with PALBI’s inclusion of platelets (a portal hypertension marker) alongside albumin and bilirubin [21,24].

In this study, key characteristics differed between the survivor (*n* = 277) and death (*n* = 224) groups. The death group had higher EZ-ALBI scores (median: −23.4 vs. −28.6 in survivors; *Z* = 11.277, *P* < 0.0001) and 3.5-fold more grade three cases (43.7 vs. 12.6%; *χ*² = 61.366, *P* < 0.0001). These findings align with known associations between impaired liver function, directly captured by EZ-ALBI via bilirubin and albumin, and cirrhosis mortality [25,26]. EZ-ALBI grade 3 correlated with severe phenotypes (higher bilirubin/INR/creatinine; lower albumin/cholinesterase), reflecting advanced multiorgan dysfunction. This aligns with a previous study showing ALBI-derived scores strongly associate with multiorgan impairment in cirrhotic patients [27]. And the primary causes of death in the deceased group were liver cirrhosis-related complications, which are consistent with the epidemiological characteristics of liver cirrhosis reported previously [2,20], reinforcing the reliability of this study regarding EZ-ALBI’s prognostic value. Notably, viral etiologies predominated in this cohort (82.6%), consistent with reports that hepatitis B virus (HBV) is the leading cause of cirrhosis in China [28]. Unlike viral cirrhosis, driven by immune-mediated hepatocyte damage and fibrosis, alcohol-related and non-alcoholic steatohepatitis -related cirrhosis involve metabolic derangements and chronic steatosis, with key pathophysiological differences that may affect prognostic marker performance [29–31]. This limits generalizability, highlighting the need for multicenter validation.

Kaplan–Meier analysis revealed that EZ-ALBI grade 3 independently predicted poorer survival (median: 29.9 vs. 65.5 months for grades 1–2; log-rank test, *P* < 0.001). Cox regression analyses confirmed this, with HR = 3.746 (univariate analysis) and 3.944 (multivariate) (95% CI: 1.772–8.777, *P* < 0.05); the wide CI likely due to the small grade 3 subgroup (133/501, 26.5%). These findings reinforce EZ-ALBI as a robust tool for liver disease risk stratification [32,33]. Furthermore, other independent risk factors included: age >58 years (cumulative organ/immune decline with aging [33]); cholinesterase ≤2962 IU/l [liver reserve surrogate tied to higher posttransjugular intrahepatic portosystemic shunt (TIPS) mortality [34]]; and neutrophil counts >4.65 × 10⁹/l (systemic inflammation driving liver damage [35]). Interestingly, ALT > 66 U/l was an independent prognostic factor (HR = 2.022, 95% CI: 1.434–2.851, *P* < 0.05), reflecting hepatocellular necrosis, distinct from EZ-ALBI’s focus on synthetic function, reinforcing EZ-ALBI’s unique value. This is notable as there was no significant baseline ALT difference between survival and death groups (*Z* = 1.761, *P* = 0.0782; Table 1). The discrepancy likely arises from the Youden index-derived cutoff (66 U/l), which enhances sensitivity to subclinical injury. Unlike median comparisons, this threshold better captures persistent hepatocellular damage, a key driver of cirrhosis, consistent with previous studies [36,37]. Collectively, these data highlight that EZ-ALBI grade 3, a core indicator of liver synthetic function, along with age (aging-related organ decline), cholinesterase (hepatic reserve), neutrophil count (systemic inflammation), and ALT (hepatocellular injury), contributes to a multidimensional prognostic framework for cirrhosis. However, EZ-ALBI has a distinct advantage: its focus on liver synthetic function (a core determinant of long-term cirrhosis outcomes) and simplicity enable rapid integration into routine risk assessment, even with adjunct factors (e.g. ALT for hepatocellular injury). This synergy, EZ-ALBI as a foundational tool complemented by other markers, may further refine risk stratification, particularly in identifying high-risk patients requiring intensified monitoring or targeted intervention.

Significantly, EZ-ALBI outperformed Child-Pugh in long-term assessments (36–60 months), likely attributable to the subjective components of Child-Pugh [6–8]; EZ-ALBI, by focusing on hepatic synthetic function (a core driver of long-term cirrhosis progression), has superior long-term stability. Conversely, MELD/MELD-Na outperformed it in the short term (6–12 months), consistent with their role in transplant allocation [10,11,13]. However, EZ-ALBI has unique utility in nontransplant settings (e.g. resource-limited hospitals), where rapid bedside assessment takes priority over transplant-specific triage. Notably, in patients with HBV-related cirrhosis complicated by SBP, a specific, severe cirrhosis complication, traditional scores (ALBI, Child-Pugh) performed suboptimally when predicting 3- and 6-month mortality compared to integrated MELD [38]. This aligns with our observation that established scores may have limited utility in specific scenarios, reinforcing the need for simpler yet robust tools like EZ-ALBI, which exhibits consistent performance across diverse follow-up periods and complex disease trajectories. Moreover, EZ-ALBI performed nearly identically to ALBI in prognostic accuracy (AUC difference ≤0.012, below the 0.05 threshold for clinical meaningfulness), corresponding to >95% consistency in individual patient prognostic judgment, consistent with previous studies [16,17]. This strong concordance supports EZ-ALBI as a viable substitute for ALBI in bedside settings, while its simplicity (no logarithmic transformations) addresses a key ALBI limitation, making it more applicable to frontline clinicians in busy or low-resource environments. PALBI (which incorporates platelets [15]) performed poorly at 36 months, suggesting long-term prognosis in cirrhotic patients tends to more on liver synthetic function (bilirubin, albumin) than portal hypertension (platelets). This aligns with a recent study showing PALBI is not a significant prognostic indicator in thrombocytopenic HCC patients, whereas ALBI (relying solely on albumin and bilirubin) retains independent predictive value [24]. Additionally, a dual-center study on cirrhotic patients with acute variceal bleeding undergoing early TIPS confirmed PALBI, ALBI, Child-Pugh, and MELD are suboptimal for predicting 1-year variceal rebleeding, highlighting the need for tailored tools in specific scenarios [39]. EZ-ALBI’s consistent performance across 6–60 months supports its utility in both short-term triage (e.g. prioritizing grade 3 patients for intensive monitoring) and long-term follow-up (e.g. adjusting surveillance frequency) across diverse clinical scenarios.

Based on the aforementioned findings, EZ-ALBI grade 3 (score ≥ −22.2) enables the identification of high-risk cirrhotic patients with shortened survival, indicating that this grading system may hold potential guiding value for the clinical management of such patients. In accordance with Chinese guidelines for the diagnosis and treatment of cirrhosis and HBV infection [19,40], clinical practice can implement a series of management strategies tailored to EZ-ALBI grade 3 patients. Regular follow-up should be conducted at 3-month intervals, involving assessments of serum albumin, bilirubin (for recalculation of the EZ-ALBI score), neutrophil count, and surveillance for HCC. For patients with refractory ascites who remain in EZ-ALBI grade 3, treatment regimens should be optimized at an early stage, such as albumin infusion combined with diuretic therapy; TIPS may also be considered when necessary. Additionally, endoscopic ligation or sclerotherapy should be performed to prevent gastrointestinal bleeding. In the context of HBV-related cirrhotic patients, long-term administration of potent nucleos(t)ide analogs with low resistance profiles (e.g. entecavir, tenofovir alafenamide) is required to achieve SVR. Furthermore, eligible patients with EZ-ALBI grade 3 should be prioritized for liver transplantation evaluation. These comprehensive measures aim to stabilize patients’ liver function, improve their EZ-ALBI grade, and optimize survival outcomes.

This study has several limitations. First, it was a single-center retrospective study, with viral-related cirrhosis accounting for 82.6% (414 cases) and nonviral cirrhosis for only 87 cases. The small sample size of nonviral cirrhosis patients would lead to insufficient statistical power, limiting the ability to perform etiology-stratified subgroup analyses and restricting the generalizability of conclusions to populations with nonviral cirrhosis. Future research should prioritize prospective, multicenter studies with large sample sizes, focusing on etiological stratification to verify the performance of the EZ-ALBI grade across different subtypes of liver cirrhosis. Second, the lack of dynamic EZ-ALBI monitoring constitutes another limitation. Given that cirrhosis is progressive, single-time-point assessments may fail to reflect treatment-related changes in liver function; future studies should therefore explore the prognostic value of serial EZ-ALBI measurements. Furthermore, the association between EZ-ALBI and cirrhosis complications (e.g. hepatic encephalopathy, HCC) remains unassessed, which limits the understanding of its utility in predicting these outcomes and necessitates further investigation.

In conclusion, in a large cirrhotic cohort, this study is the first to systematically validate that EZ-ALBI maintains stable prognostic value over 6–60 months, with long-term performance surpassing Child-Pugh. These findings establish it as a novel risk stratification tool for liver cirrhosis, differentiating it from its role in HCC. As a simple, objective, and robust tool, EZ-ALBI bolsters clinical utility via strong correlation with established models, independent predictive value, and consistent short- and long-term performance. Notably, its freedom from logarithmic transformations significantly shortens calculation time vs. ALBI, greatly enhancing feasibility for real-time bedside risk stratification, a key clinical advantage.

## Acknowledgements

This research did not receive any specific grant from funding agencies in the public, commercial, or not-for-profit sectors.

J.L. conceived the study, curated the data, developed the methodology, handled software, validated the data, and wrote the original draft. Y.-s.W. supervised the study and reviewed and edited the manuscript.

The figures supporting the study findings are incorporated in the manuscript, and the original datasets can be obtained from the first author or the corresponding author upon reasonable request.

The authors confirm that this manuscript has not been published elsewhere and is not under consideration by any other journal.

### Conflicts of interest

There are no conflicts of interest.
